# Chidamide combined with decitabine, venetoclax, and low-dose cytarabine for relapsed/refractory acute myeloid leukemia: a single-center case series

**DOI:** 10.3389/fonc.2026.1760614

**Published:** 2026-02-24

**Authors:** Qing Li, Jie Ji, Yu Wu

**Affiliations:** Department of Hematology and Institute of Hematology, West China Hospital, Sichuan University, Chengdu, Sichuan, China

**Keywords:** case series, chidamide, combination therapy, epigenetic therapy, relapsed/refractory acute myeloid leukemia (R/R AML), single-center study, venetoclax

## Abstract

Relapsed/refractory acute myeloid leukemia (R/R AML) carries a poor prognosis. We report three patients with R/R AML, each of whom had relapsed after at least two prior chemotherapy regimens or hematopoietic stem-cell transplantation (HSCT), treated with chidamide, decitabine, venetoclax, and low-dose cytarabine (LDAC) (CHI-DEC-VEN-LDAC). After a single treatment cycle, all three patients achieved complete remission (CR), and two attained minimal residual disease (MRD) negativity. Adverse events were manageable, primarily consisting of cytopenias and infections, with no treatment-related deaths. These results suggest that the therapy is effective and well-tolerated, particularly in patients with R/R AML who have had extensive prior treatment. The regimen shows promise as a potential bridging or debulking strategy, but further research with larger cohorts and longer follow-up is needed to confirm its long-term efficacy. These cases highlight the potential benefits of chemotherapy-free or low-chemotherapy approaches for R/R AML treatment.

## Introduction

Relapsed/refractory acute myeloid leukemia (R/R AML) remains a major clinical challenge with limited effective options and poor prognosis. Conventional salvage chemotherapy often yields suboptimal responses and substantial toxicity, particularly in patients with adverse cytogenetic or molecular features. Recent advances in epigenetic modulators and apoptosis-targeted therapies have opened new avenues for treatment (1). Chidamide, a selective histone deacetylase inhibitor, can enhance the activity of hypomethylating agents such as decitabine by reprogramming gene expression and promoting apoptosis, thereby exerting synergistic anti-leukemic effects (2). In R/R AML, chidamide–decitabine–based regimens have produced overall response rates of approximately 42.9–46.2% (3, 4). Venetoclax, a selective BCL-2 inhibitor, further improves outcomes when combined with hypomethylating agents or low-dose cytarabine (LDAC); in the R/R setting, venetoclax-based regimens have achieved overall response rates up to 68%, with complete remission/complete remission with incomplete hematologic recovery (CR/CRi) rates around 53% and a median overall survival of 13.1 months (5). Building on these insights, the four-drug combination of chidamide, decitabine, venetoclax, and LDAC (CHI-DEC-VEN-LDAC) represents a mechanism-driven therapeutic strategy. Here, we present three cases of R/R AML treated with this regimen, all of whom achieved CR.

## Case description

### Case 1

A 49-year-old woman presented in August 2022 with fatigue and gingival swelling and was diagnosed with acute monocytic leukemia. Cytogenetics showed a normal karyotype (46,XX[20]); targeted sequencing detected DNMT3A, IDH2, NF1, and NRAS mutations, classifying her as intermediate-risk per NCCN 2021 guidelines (**Table 1**). She received two induction cycles with DA (daunorubicin plus cytarabine) with decitabine priming, achieving CR with minimal residual disease (MRD) positivity (0.3%). Induction was complicated by *Pseudomonas aeruginosa* sepsis. After one cycle of intermediate-dose cytarabine consolidation, MRD negativity was achieved, followed by a second consolidation with HA (homoharringtonine plus cytarabine).

**Table 1:** Cytogenetic and molecular characteristics of three patients.

N0.	Age/Gender	Chromosome Karyotype	Molecular characteristics	relapses	transplant	treatment regimen	response
P1	49/F	46,XX[20]	DNMT3A,	2	No	Chi+Ven+Dec+A	CR, MRD-
			IDH2, NF1,				
			NRAS				
P2	48/M	NA	CBFβ/MYH11,	3	Yes	Chi+Ven+Dec+A	CR, MRD-
			FLT3-TKD				
P3	41/F	39-40, XX, -5, -7, ins (11;?) (q13;? ), -12, -13, i (14)	CEBPA, TP53,	1	No	Chi+Ven+Dec+A	CR, MRD+
		(q10), -16, -20, -22, +mar, inc [cp17]/46, XX [3]	PNF6, PTPN1				

In March 2023, she was admitted for planned allogeneic hematopoietic stem-cell transplantation (HSCT). Pre-transplant marrow showed MRD negativity, clearance of IDH2 and DNMT3A mutations, and a WT1 level of 10.43%. She subsequently received two additional high-dose cytarabine (HD-Ara-C) cycles (March and May) and a fifth cycle with venetoclax plus azacitidine in July 2023; MRD remained negative. She also received three prophylactic intrathecal treatments, with normal cerebrospinal fluid throughout. In October 2023, bone marrow examination confirmed relapsed AML, with IDH2 exon 4 mutation re-emergence and WT1 3.52%. The patient declined enasidenib and received FLAG (fludarabine, cytarabine, G-CSF) re-induction, complicated by recurrent infections. In November, bone marrow showed CR with MRD negativity.

**Figure 1:**
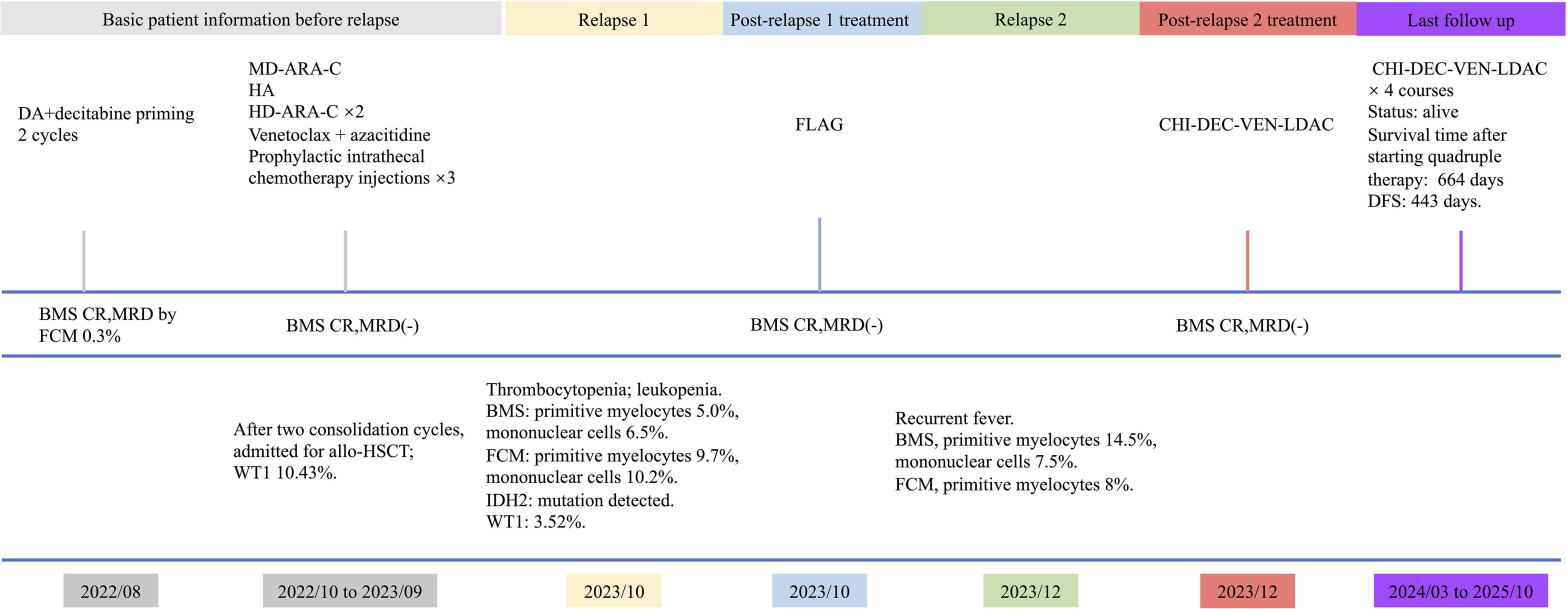
Timeline of disease course of patient 1. BMS, bone marrow smear; FCM, flow cytometry; DFS, disease-free survival.

She was rehospitalized in December for recurrent fever and refractory infection, and marrow reassessment documented a second relapse. Re-induction with CHI-DEC-VEN-LDAC (chidamide 30 mg twice weekly; venetoclax 100 mg daily for 14 days; decitabine 10 mg daily for 7 days; low-dose cytarabine 25 mg twice daily for 7 days) commenced in January 2024. Fever transiently resolved, recurred during myelosuppression, and was controlled with anti-infectives. She achieved CR with MRD negativity after this cycle. From March through December 2024, the patient completed four cycles of CHI-DEC-VEN-LDAC consolidation, maintaining CR with MRD negativity throughout. Relapse was documented on April 16, 2025. As of October 18, 2025, the patient remains alive. The clinical timeline is summarized in **Figure 1**.

### Case 2

A 45-year-old man presented in June 2020 with oral ulcers, bleeding, and skin ecchymoses. Laboratory tests revealed anemia (Hb 101 g/L), severe thrombocytopenia (platelets 7×10^9^/L), leukocytosis (WBC 97.94×10^9^/L), and 61% blasts. Bone marrow analysis confirmed AML M4 with CBFB/MYH11 fusion and FLT3-TKD mutation, classifying him as intermediate-risk according to the NCCN 2021 guidelines (**Table 1**). After IA induction, he achieved CR with MRD positivity (0.3%). Following consolidation with IDA plus HD-Ara-C, midostaurin plus homoharringtonine (HHT) plus HD-Ara-C, and IA, autologous peripheral stem cells were collected (April 2021), and interleukin-2 maintenance was initiated. In April 2021, chest CT revealed mass-like consolidation consistent with *Aspergillus* pneumonia, delaying autologous transplantation; antifungal therapy was started.

He relapsed in October 2021 and received FLAG re-induction, achieving CR with MRD negativity. In December 2021, consolidation with decitabine plus HAG (homoharringtonine, cytarabine, G-CSF) was administered. By February 2022, bone marrow showed CR with MRD negativity and a CBFB/MYH11/ABL1 ratio of 0.02%. No fully matched donor was available in the China Marrow Donor Program. Haploidentical HSCT from his son was recommended but declined; He chose autologous HSCT. In March 2022, autologous HSCT was performed after Chi-FAB (chidamide, fludarabine, Ara-C, and busulfan) conditioning, with 15×10^5^ CD34+ cells/kg reinfused. Post-transplant marrow confirmed remission. Maintenance included interleukin-2 and NK-cell infusions. Marrow examinations in April and August 2022 showed no residual leukemia, with CBFB/MYH11 and FLT3-TKD negative.

A second relapse occurred in November 2022 (CBFB/MYH11 positive, WT1 high). Re-induction with venetoclax, azacitidine, and HHT began in December; no matched donor was identified (brother 11/12 HLA). By February 2023, bone marrow showed no residual leukemia. Haploidentical HSCT was again recommended but declined; the patient chose consolidation chemotherapy. The first course (venetoclax, azacitidine, cytarabine, HHT) began in March with remission confirmed in April; a second similar course was given in June. He then discontinued further therapy while remaining in hematologic remission.

**Figure 2:**
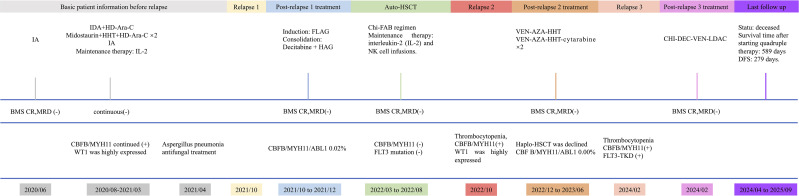
Timeline of disease course of patient 2. BMS, bone marrow smear; FCM, flow cytometry; DF, disease-free survival.

In February 2024, thrombocytopenia recurred with 40% circulating blasts. Bone marrow confirmed a third relapse with CBFB/MYH11 positivity and FLT3–TKD mutation. Re-induction with CHI-DEC-VEN-LDAC (chidamide 30 mg twice weekly; venetoclax 200 mg daily for 14 days; decitabine 10 mg daily for 7 days; low-dose cytarabine 25 mg twice daily for 7 days) was started in February. Febrile neutropenia was managed with empiric antibacterials and antifungals; blood cultures yielded ESBL-producing *Escherichia coli*. Subsequent marrow evaluation demonstrated CR with MRD negativity. However, the patient’s disease relapsed on January 4, 2025, and he received alternative treatment regimens, which did not achieve CR. Ultimately, he passed away on September 7, 2025. The clinical timeline is shown in **Figure 2**.

### Case 3

A 40-year-old woman was diagnosed with AML in August 2023 after presenting with thrombocytopenia and anemia. The diagnosis was confirmed by peripheral blood tests, bone marrow smear, flow cytometry, and biopsy. Targeted sequencing of 248 myeloid genes detected mutations in CEBPA, TP53, PNF6, and PTPN11. Karyotyping showed a complex abnormal profile—39–40,XX, −5, −7, ins (11);?(q13);?, −12, −13, i (14)(q10), −16, −20, −22, +mar, inc [cp17]/46,XX[3]—classifying her as high-risk per NCCN 2021 guidelines (**Table 1**). Induction chemotherapy with venetoclax, cytarabine, and daunorubicin was initiated in August 2023. In September, bone marrow evaluation showed CR with MRD positivity. In October, she received the first consolidation course with venetoclax plus HD-Ara-C.

**Figure 3:**
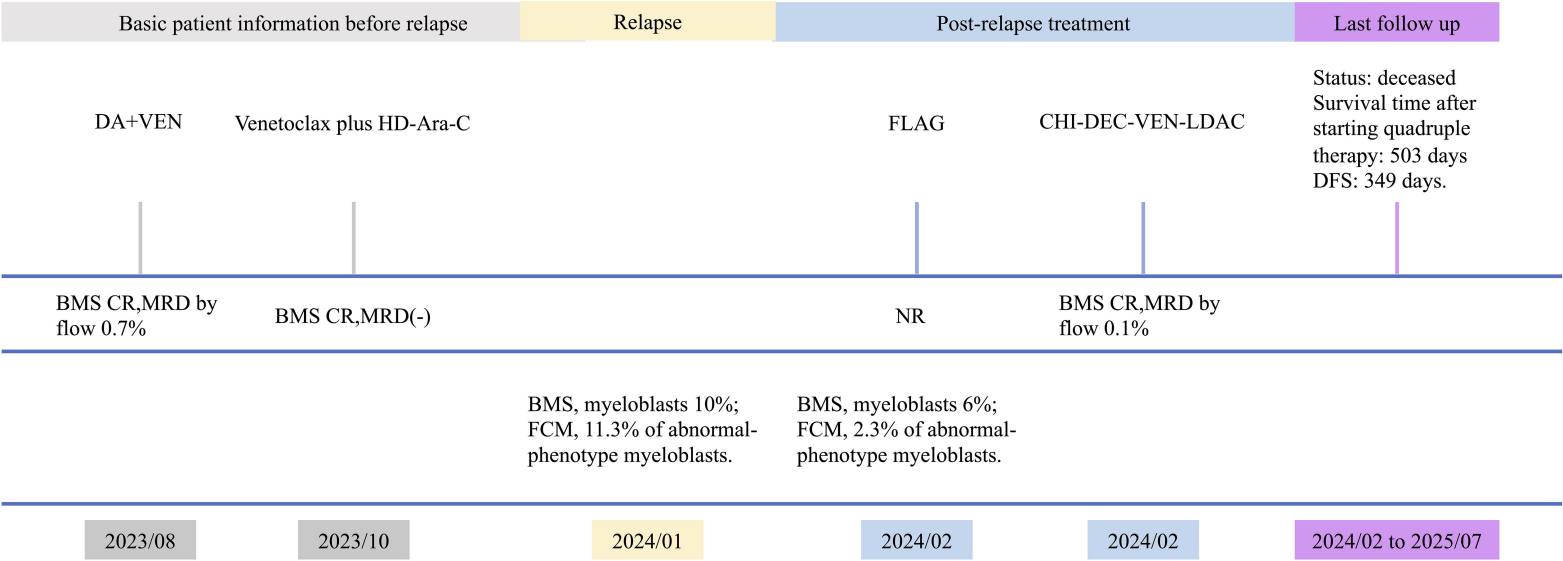
Timeline of disease course of patient 3. BMS, bone marrow smear; FCM, flow cytometry; NR, no remission; DFS, disease-free survival.

A fully matched unrelated donor was identified through the China Marrow Donor Program, and she was admitted for allogeneic HSCT in January 2024. However, bone marrow re-examination that month indicated relapsed AML. Re-induction with a reduced-dose FLAG regimen was started due to transfusion constraints. In February 2024, the bone marrow showed no remission. Given the adverse prognosis associated with TP53 mutation, options including CAR-T therapy followed by transplantation were discussed; the patient elected to continue chemotherapy. Re-induction with CHI-DEC-VEN-LDAC began in February 2024 (chidamide 20 mg twice weekly; venetoclax 200 mg daily ×14 days; decitabine 10 mg daily ×7 days; low-dose cytarabine 25 mg twice daily ×7 days). The patient developed febrile neutropenia and was treated with antibiotics. In March 2024, bone marrow smear and flow cytometry showed CR with positive MRD. The patient subsequently underwent allogeneic HSCT. Approximately 10 months after the transplant, the disease relapsed, and the patient passed away in July 2025. The clinical timeline is depicted in **Figure 3**.

## Discussion

Despite therapeutic advances, relapse remains the leading cause of death in AML (6). Up to 10–40% of younger patients and 40–60% of older adults fail to respond to frontline therapy (7). Even among patients who attain CR and proceed to hematopoietic stem-cell transplantation, relapse rates approach 40% (8). Consequently, R/R AML remains a major clinical challenge. Re-induction chemotherapy yields modest outcomes, with CR rates of 20–60%, and durable remissions are uncommon with standard salvage regimens (9, 10). These realities underscore the urgent need for more effective and better-tolerated therapies.

This case series is among the first to describe the CHI-DEC-VEN-LDAC regimen in R/R AML patients. All three patients had failed at least two prior lines of therapy and exhibited intermediate-to-high-risk features. Notably, all achieved complete remission after one or two cycles, highlighting the promising antileukemic activity of this regimen in a difficult-to-treat population.

Previous studies demonstrated the efficacy of the CDCAG regimen (chidamide, decitabine, cytarabine, aclarubicin, and G-CSF) achieved an overall response rate (ORR) of 46.2% in R/R AML (3). Adding venetoclax to CDCAG (CDCAG-VEN) further improved outcomes—achieving a higher response rate (78.6% vs. 45.5%) and better survival—without excess toxicity (11). Our regimen, however, excludes aclarubicin, avoiding its associated cardiotoxicity and myelosuppression. Additionally, we use a lower dose of cytarabine (25 mg twice daily for 7 days) compared to higher doses used in other studies [e.g., 50 mg/m²/day (3) or 75–100 mg/m² twice daily (11)]. This reduction minimizes myelosuppression—a common side effect in AML treatment—and aims for a better balance between efficacy and safety, lowering the risk of bone marrow failure.

MRD negativity is now recognized as a meaningful endpoint in both newly diagnosed and R/R AML, correlating with reduced relapse risk before and after transplantation (12–15). In our cohort, 2 out of 3 patients (66.7%) achieved MRD-negative CR, underscoring the potential of this regimen to induce deep remissions. The third patient, who had complex cytogenetics and a TP53 mutation, did not achieve MRD negativity but exhibited a marked reduction in disease burden to 0.1% by flow cytometry.

Venetoclax is a selective BCL-2 inhibitor that triggers apoptosis by restoring mitochondrial outer-membrane permeabilization and activating caspase cascades (16). It also disrupts mitochondrial metabolism—including oxidative phosphorylation—and perturbs reactive oxygen species homeostasis, thereby targeting both bulk AML cells and leukemia stem cells (17, 18). In combination with hypomethylating agents such as azacitidine or decitabine, venetoclax has shown substantial clinical efficacy in newly diagnosed elderly or unfit AML patients (19–21). Resistance, however, can emerge; a key mechanism is a shift toward dependence on the anti-apoptotic protein MCL-1, which enables leukemic cells to bypass BCL-2 inhibition and evade apoptosis (22). Strategies to suppress MCL-1—such as inhibitors of deubiquitinases, CDK9, or SPHK1—offer potential avenues to overcome venetoclax resistance (23–25). Although CDK9 inhibitors are furthest along clinically, benefit in venetoclax-resistant AML is not yet established.

Chidamide exerts antileukemic effects by inducing DNA double-strand breaks and modulating apoptotic signaling, downregulating MCL-1 and BCL-XL while upregulating the pro-apoptotic protein BIM (26). Chidamide also synergizes with cytarabine to enhance apoptosis, particularly in FLT3-ITD–positive and relapsed/refractory AML models, supporting a biologically plausible combination strategy (27).

All three patients in this case series tolerated the CHI-DEC-VEN-LDAC regimen well, with the main side effects being transient cytopenia and manageable infections; no treatment-related deaths occurred. While the cohort was small, the safety profile is promising for patients with limited tolerance for intensive chemotherapy, including those with active or recent infections. In conclusion, the CHI-DEC-VEN-LDAC regimen appears to be an effective bridging or debulking therapy for R/R AML, demonstrating a high complete response rate, significant MRD negativity, and manageable toxicity. This supports the concept that chemotherapy-free or low-dose regimens can preserve antileukemic efficacy while reducing treatment-related toxicity, thereby offering a potential clinical advantage. However, given the limitations of this study, these conclusions should be interpreted with caution.

This study has several limitations. First, its retrospective design and small sample size limit the generalizability of the results. Second, although all reported patients achieved complete remission, the absence of non-responders raises the possibility of selection bias and precludes a comprehensive evaluation of treatment efficacy and safety across a broader patient population. In addition, the relatively limited follow-up duration restricts conclusions regarding long-term outcomes. Larger prospective studies are therefore warranted. An ongoing registered clinical trial (ChiCTR2300073732) is expected to provide more robust evidence to further validate these findings.

## Data Availability

The original contributions presented in the study are included in the article/supplementary material. Further inquiries can be directed to the corresponding authors.
